# Microbiological Profile and Antimicrobial Resistance Patterns in an Intensive Care Unit: Emphasis on ESKAPE Pathogens

**DOI:** 10.3390/antibiotics15050500

**Published:** 2026-05-17

**Authors:** Leandro Aparecido de Souza, Jéssica Cristina Bilizario Noguerol Andrade, Fernando de Sá Del Fiol

**Affiliations:** Doctoral Program, Pharmaceutical Sciences, University of Sorocaba, Sorocaba 18023-000, SP, Brazil; leandro.souza@prof.uniso.br (L.A.d.S.); jessica.andrade@prof.uniso.br (J.C.B.N.A.)

**Keywords:** antimicrobial resistance, intensive care unit, multidrug resistance, ESKAPE, antimicrobial stewardship

## Abstract

**Background/Objectives:** Antimicrobial resistance (AMR) is a major global public health threat, particularly in intensive care units (ICUs), where critically ill patients are exposed to high antimicrobial pressure. ESKAPE pathogens play a central role in healthcare-associated infections and are frequently associated with multidrug resistance. This study aimed to evaluate the microbiological profiles and antimicrobial resistance patterns of bacterial isolates from an adult ICU in a tertiary hospital in Brazil. **Methods:** A retrospective, observational, cross-sectional study was conducted using microbiological culture data from patients admitted during 2024 to the adult intensive care unit of a tertiary hospital in Brazil. Bacterial isolates from clinical specimens were included, and antimicrobial susceptibility testing was performed according to routine laboratory procedures. Descriptive statistics were used to summarize microorganism frequency and antimicrobial resistance rates. **Results:** A total of 1869 isolates were analyzed, with predominance of Gram-negative bacteria. The most frequent pathogens were *Klebsiella pneumoniae* (287/1869; 15.33%), *Staphylococcus haemolyticus* (164/1869; 8.76%), *Enterococcus faecalis* (144/1869; 7.69%), and *Acinetobacter baumannii* (135/1869; 7.21%). A high proportion of isolates belonged to the ESKAPE group. Gram-negative bacteria, particularly *Klebsiella pneumoniae* and *Acinetobacter baumannii*, showed high resistance to β-lactams, cephalosporins, carbapenems, and quinolones. Among Gram-positive organisms, resistance was high for macrolides, oxacillin, and clindamycin, while glycopeptides and linezolid remained effective. **Conclusions:** These findings highlight the importance of continuous microbiological surveillance and robust antimicrobial stewardship strategies. The present study adds novel local epidemiological evidence from a Brazilian ICU by integrating species-level distribution, antimicrobial-specific resistance, and resistance patterns by pharmacological class, with particular emphasis on ESKAPE pathogens.

## 1. Introduction

Antimicrobial resistance (AMR) is currently recognized as one of the greatest challenges to global public health, compromising the therapeutic effectiveness of drugs widely used to treat bacterial infections. This phenomenon occurs when microorganisms develop mechanisms capable of neutralizing or reducing the activity of antimicrobials that were previously effective, resulting in therapeutic failure, increased morbidity and mortality, and higher healthcare costs [1]. The progression of AMR is closely associated with the inappropriate and excessive use of antibiotics in different healthcare settings, which promotes the selection of resistant strains and the dissemination of these pathogens in hospital environments.

Over recent decades, the rise of bacterial resistance has been widely documented worldwide, leading to the creation of global surveillance systems to monitor antimicrobial consumption and resistance patterns. Among these initiatives, the Global Antimicrobial Resistance and Use Surveillance System (GLASS), developed by the World Health Organization, stands out as an important strategy aimed at standardizing data collection and guiding global strategies to combat antimicrobial resistance [2]. Through these initiatives, it has become possible to better understand the dynamics of resistance dissemination and to support the development of public policies aimed at rational antimicrobial use.

The hospital environment, particularly Intensive Care Units (ICUs), represents a critical setting for the emergence and spread of resistant microorganisms. Patients admitted to these units often present severe clinical conditions, require invasive procedures, and frequently receive multiple broad-spectrum antimicrobials, factors that favor the selection of multidrug-resistant pathogens [3,4]. Furthermore, the high density of therapeutic interventions and the complexity of intensive care contribute to the circulation and persistence of these microorganisms in hospital settings.

Among the pathogens of greatest clinical relevance in hospital settings are those belonging to the ESKAPE group, which includes species such as *Staphylococcus aureus*, *Klebsiella pneumoniae*, *Acinetobacter baumannii*, *Pseudomonas aeruginosa*, and *Enterobacter* spp. These microorganisms are recognized for their ability to develop multiple resistance mechanisms and for their frequent association with healthcare-associated infections, particularly among critically ill patients [5,6]. The dissemination of these multidrug-resistant bacteria has a significant impact on clinical outcomes, making the selection of effective antimicrobial therapies increasingly challenging.

The selective pressure exerted by intensive antimicrobial use also plays a central role in the evolution of bacterial resistance. Inappropriate antibiotic use, including empirical prescriptions without microbiological confirmation or prolonged administration of broad-spectrum agents, contributes to changes in the hospital microbial ecology and promotes the emergence of resistant strains [1]. This phenomenon has been frequently observed in intensive care units, where antimicrobial consumption is substantially higher compared with other hospital sectors.

In this context, the implementation of antimicrobial stewardship programs has been widely recommended as a strategy to promote the rational use of these medications. These programs aim to optimize antimicrobial selection, dosage, treatment duration, and route of administration, thereby improving clinical outcomes and reducing the emergence of antimicrobial resistance [7,8]. Evidence indicates that the adoption of such strategies can result in clinical, epidemiological, and economic benefits for healthcare systems.

Despite advances in policies for antimicrobial resistance control and surveillance, significant gaps remain in the understanding of microbiological profiles and resistance patterns across different healthcare institutions. Studies conducted in ICUs have demonstrated substantial variability in the distribution of microorganisms and resistance profiles, reflecting local epidemiological characteristics and specific healthcare practices [9,10]. Therefore, knowledge of the institutional microbiological profiles is essential to guide therapeutic protocols and infection control strategies.

Given this scenario, analyzing the microbiological profile and antimicrobial resistance patterns in intensive care units becomes essential to support clinical decision-making and therapeutic management strategies. Understanding these characteristics allows the identification of predominant microorganisms, monitoring of resistance trends, and support for the development of evidence-based institutional protocols, contributing to the rational use of antimicrobials and improving the quality of care for critically ill patients [11].

The predominance of these pathogens is consistent with their well-established role as leading causes of healthcare-associated infections in critically ill patients. ESKAPE organisms are characterized by their remarkable ability to evade the effects of antimicrobial agents and to persist in hospital environments, particularly in intensive care settings where antimicrobial exposure is high [5,6].

The aim of this study was to evaluate the microbiological profiles and antimicrobial resistance patterns of pathogens isolated from patients admitted to an adult Intensive Care Unit in a large tertiary hospital in Brazil. Additionally, the study sought to characterize the distribution of the most prevalent microorganisms and analyze their resistance patterns to commonly used antimicrobial agents, providing local epidemiological data to support clinical decision-making and strategies for the rational use of antimicrobials in critically ill patients.

## 2. Results

### 2.1. Patient Demographics

During the study period, the ICU population was predominantly composed of older adults, with the highest concentration of patients in the 70–79-year age group. Mortality was higher among older patients, while no relevant difference in clinical outcome was observed according to sex.

### 2.2. Microbiological Distribution of Isolates in the ICU

A total of 1869 microbial isolates were identified during the study period (Table 1). Gram-negative bacteria predominated, especially *Klebsiella pneumoniae*, *Acinetobacter baumannii*, *Pseudomonas aeruginosa*, and *Escherichia coli*. Among Gram-positive organisms, the most frequent isolates were *Staphylococcus haemolyticus*, *Enterococcus faecalis*, *Staphylococcus hominis*, and *Staphylococcus aureus*.

*Klebsiella pneumoniae* was the most prevalent microorganism, accounting for 287/1869 isolates (15.33%), followed by *Staphylococcus haemolyticus* (164/1869; 8.76%), *Enterococcus faecalis* (144/1869; 7.69%), and *Acinetobacter baumannii* (135/1869; 7.21%). Other frequently isolated microorganisms included *Staphylococcus epidermidis*, *Pseudomonas aeruginosa*, *Escherichia coli*, and *Staphylococcus aureus*.

### 2.3. Antimicrobial Resistance Profiles Among Gram-Negative Bacteria

The antimicrobial resistance profiles of Gram-negative bacteria are summarized in Table 2. *Klebsiella pneumoniae* demonstrated elevated resistance rates to several β-lactam agents, including cefepime (91.1%), ceftazidime (90.1%), ceftriaxone (93.9%), and amoxicillin–clavulanate (88.1%). Resistance to carbapenems was also substantial, with rates of 70.8% for ertapenem and 67.6% for meropenem.

*Acinetobacter baumannii* exhibited resistance rates exceeding 96% for carbapenems and piperacillin–tazobactam. High resistance levels were also observed for aminoglycosides and trimethoprim–sulfamethoxazole. *Pseudomonas aeruginosa* showed lower resistance rates compared with other Gram-negative organisms, although resistance to piperacillin–tazobactam (39.3%) and carbapenems remained clinically relevant.

Among Enterobacterales, *Escherichia coli* presented elevated resistance to cephalosporins and quinolones, whereas *Proteus mirabilis* demonstrated high resistance rates to quinolones, cephalosporins, and trimethoprim–sulfamethoxazole. Resistance profiles of *Enterobacter cloacae*, *Enterobacter hormaechei*, and *Serratia marcescens* varied according to the antimicrobial agent tested.

### 2.4. Antimicrobial Resistance Profiles Among Gram-Positive Bacteria

The resistance profiles of Gram-positive bacteria are presented in Table 3. Coagulase-negative staphylococci represented the predominant Gram-positive microorganisms and showed elevated resistance rates to oxacillin, erythromycin, and clindamycin. *Staphylococcus haemolyticus* exhibited resistance rates of 98.5% to erythromycin, 97.8% to oxacillin, and 92.2% to clindamycin. Similarly, *Staphylococcus epidermidis* demonstrated resistance rates of 93.4% to oxacillin and 87.3% to erythromycin. *Staphylococcus aureus* showed lower resistance rates overall, although resistance to erythromycin and oxacillin remained relevant. Resistance to glycopeptides and linezolid remained low among most Gram-positive isolates. However, *Enterococcus faecium* demonstrated elevated resistance to ampicillin and quinolones, whereas *Enterococcus faecalis* showed comparatively lower resistance rates.

### 2.5. Resistance Patterns by Antimicrobial Class

The resistance profiles according to antimicrobial class are summarized in Table 4. Among Gram-negative organisms, particularly *Acinetobacter baumannii* and *Klebsiella pneumoniae*, high resistance rates were observed for penicillins, cephalosporins, carbapenems, and quinolones.

*Acinetobacter baumannii* demonstrated resistance rates exceeding 96% for penicillins, carbapenems, and quinolones. *Klebsiella pneumoniae* presented resistance rates of 91.5% to penicillins, 79.8% to cephalosporins, and 69.2% to carbapenems. In contrast, *Pseudomonas aeruginosa* showed comparatively lower resistance rates to several antimicrobial classes.

Among Gram-positive bacteria, coagulase-negative staphylococci demonstrated elevated resistance to macrolides, quinolones, and penicillins. Resistance to glycopeptides and oxazolidinones remained low among most Gram-positive isolates.

To contextualize the resistance rates observed in this study, selected resistance patterns were compared descriptively with previously published ICU surveillance studies and international reports. Given the absence of patient-level comparative data and the ecological nature of the available benchmarks, no inferential statistical test was applied between external studies. Instead, comparisons were performed by evaluating absolute differences in resistance proportions between the present ICU isolates and published resistance ranges reported in the literature. This approach was adopted to avoid inappropriate statistical inference across heterogeneous populations, microbiological methods, and surveillance periods.

### 2.6. ESKAPE Pathogens: Epidemiological Relevance and Resistance Profiles

A substantial proportion of the isolates identified in the ICU belonged to the ESKAPE group, including *Klebsiella pneumoniae*, *Acinetobacter baumannii*, *Pseudomonas aeruginosa*, *Staphylococcus aureus*, and *Enterococcus* spp. These microorganisms demonstrated resistance involving multiple antimicrobial classes, particularly among Gram-negative pathogens. High resistance rates to β-lactams, carbapenems, and quinolones were observed among *Klebsiella pneumoniae* and *Acinetobacter baumannii*. Among Gram-positive ESKAPE pathogens, Enterococcus faecium demonstrated high resistance rates to clinically important agents, including ampicillin, levofloxacin, vancomycin, and teicoplanin, whereas Staphylococcus aureus exhibited relevant resistance to oxacillin, erythromycin, and clindamycin, suggesting the persistence of multidrug-resistant Gram-positive organisms in the ICU setting.

## 3. Discussion

The present study demonstrated a high burden of multidrug-resistant microorganisms in the ICU setting, with predominance of Gram-negative bacteria, particularly *Klebsiella pneumoniae* and *Acinetobacter baumannii*. These findings are consistent with previous investigations showing that ICUs represent critical environments for the emergence and dissemination of resistant pathogens due to intense antimicrobial exposure, prolonged hospitalization, and frequent use of invasive devices [3,4,12].

A substantial proportion of isolates identified in the present study belonged to the ESKAPE group, reinforcing the epidemiological relevance of these pathogens in healthcare-associated infections. ESKAPE organisms are recognized for their remarkable ability to evade antimicrobial activity and accumulate resistance determinants, representing one of the greatest challenges in modern hospital medicine [5,6]. Similar microbiological distributions have been described in ICU surveillance studies conducted in Europe, Latin America, and Asia [9,10,12].

The predominance of *Klebsiella pneumoniae* among ICU isolates is particularly concerning due to its elevated resistance rates to cephalosporins, carbapenems, and quinolones. Resistance levels observed for cefepime, ceftazidime, ceftriaxone, and carbapenems suggest the dissemination of extended-spectrum β-lactamase (ESBL)- and carbapenemase-producing strains in the ICU environment [13,14]. Similar findings have been reported in studies evaluating multidrug-resistant Gram-negative bacilli in critically ill patients [11,13].

*Acinetobacter baumannii* exhibited one of the most critical resistance profiles observed in the study, with resistance rates exceeding 96% for carbapenems and quinolones. This profile is compatible with carbapenem-resistant *Acinetobacter baumannii* (CRAB), a pathogen classified as a critical priority microorganism by international health agencies [15,16]. The persistence of highly resistant *A. baumannii* strains in ICUs has been associated with selective antimicrobial pressure, environmental persistence, and efficient acquisition of resistance determinants [16,17].

Although *Pseudomonas aeruginosa* showed comparatively lower resistance rates than *Klebsiella pneumoniae* and *Acinetobacter baumannii*, resistance to piperacillin–tazobactam and carbapenems remained clinically relevant. Previous studies have demonstrated that *P. aeruginosa* possesses intrinsic and acquired resistance mechanisms capable of substantially limiting therapeutic options in critically ill patients [13].

Among Gram-positive organisms, coagulase-negative staphylococci demonstrated elevated resistance to oxacillin, erythromycin, and clindamycin. These findings suggest a high prevalence of methicillin-resistant coagulase-negative staphylococci in the ICU environment, microorganisms frequently associated with catheter-related infections and bloodstream infections [18]. In contrast, glycopeptides and linezolid maintained high in vitro activity against most Gram-positive isolates, indicating that these agents remain important therapeutic options for severe Gram-positive infections.

The resistance profile of *Enterococcus faecium* deserves particular attention due to the elevated resistance observed for ampicillin and quinolones. Multidrug-resistant enterococci have become increasingly important in hospital settings, especially among critically ill patients exposed to prolonged antimicrobial therapy [18]. Conversely, *Enterococcus faecalis* demonstrated comparatively lower resistance rates, a finding consistent with previous epidemiological studies.

The analysis according to antimicrobial class demonstrated widespread resistance among Gram-negative organisms to penicillins, cephalosporins, carbapenems, and quinolones. These findings substantially compromise empirical therapeutic strategies in ICU settings and reinforce the importance of continuous microbiological surveillance [11,19]. In contrast, resistance to glycopeptides and oxazolidinones remained low among Gram-positive organisms, preserving the clinical utility of these antimicrobial classes.

The resistance profiles observed in the present study may also reflect the high antimicrobial selective pressure commonly found in intensive care environments. Previous investigations conducted in the same institution demonstrated elevated consumption of broad-spectrum antimicrobial agents before implementation of antimicrobial stewardship interventions [7]. High antimicrobial exposure contributes directly to the selection and persistence of multidrug-resistant microorganisms, particularly among ESKAPE pathogens [17,20].

Taken together, these findings reinforce the importance of local epidemiological surveillance to guide empirical antimicrobial therapy and support institutional antimicrobial stewardship programs. The high burden of multidrug-resistant organisms identified in this ICU underscores the need for continuous infection control measures, rational antimicrobial use, and implementation of evidence-based therapeutic protocols to mitigate the impact of antimicrobial resistance in critically ill patients [7,11,17].

## 4. Materials and Methods

### 4.1. Study Design and Setting

This study was designed as a retrospective, observational, cross-sectional study conducted in the adult Intensive Care Unit (ICU) of a large tertiary hospital located in the state of São Paulo, Brazil. The ICU provides care for critically ill adult patients with a wide range of medical and surgical conditions and represents a high-complexity environment with frequent use of invasive procedures and antimicrobial therapy. Intensive care units are recognized as hospital settings with high antimicrobial consumption and a significant burden of healthcare-associated infections, factors that contribute to the emergence and dissemination of antimicrobial-resistant microorganisms [20,21].

### 4.2. Participants

The study was conducted in a large tertiary hospital located in the city of Sorocaba, state of São Paulo, southeastern Brazil. Sorocaba has an estimated population of approximately 762,172 inhabitants and represents one of the largest urban and healthcare centers in the state of São Paulo. The hospital is an important regional referral center providing tertiary care for a large surrounding population. The adult ICU has approximately 50 beds and admits critically ill medical and surgical patients, representing a high-complexity environment with frequent use of invasive devices and broad-spectrum antimicrobials. Inclusion criteria comprised all bacterial isolates recovered from clinical specimens collected from ICU patients between January and December 2024. Duplicate isolates from the same infectious episode were carefully evaluated to avoid overrepresentation. Fungal isolates were included only in the microbiological distribution analysis (Table 1) but were excluded from the antimicrobial resistance analysis, which focused exclusively on bacterial pathogens.

No formal sample size calculation was performed because this was a retrospective surveillance study based on all eligible microbiological cultures obtained during the study period. Therefore, the sample size was defined by the total number of bacterial isolates recovered from ICU patients between January and December 2024. This census-based approach was considered appropriate because the objective was to characterize the local microbiological and resistance profiles rather than to test a predefined intervention effect.

### 4.3. Data Sources and Collection

Data were collected retrospectively from the hospital’s electronic medical record system and microbiology laboratory database. The following information was extracted from the laboratory records: microorganism species identified in culture; antimicrobial susceptibility results; antimicrobial agents tested against each isolate. All data were anonymized prior to analysis to ensure patient confidentiality.

### 4.4. Microbiological Identification

Microorganism identification was performed by the hospital clinical microbiology laboratory using routine diagnostic methods applied in clinical practice. Isolates were classified according to species and microbiological characteristics, including Gram staining. Particular attention was given to pathogens frequently associated with healthcare-associated infections and antimicrobial resistance, including members of the ESKAPE group, which are recognized as clinically relevant pathogens responsible for a significant proportion of severe hospital infections [22,23].

### 4.5. Antimicrobial Susceptibility Testing

Antimicrobial susceptibility testing was performed for each bacterial isolate according to the routine procedures adopted by the clinical microbiology laboratory. The susceptibility profiles of each microorganism was determined against a panel of commonly used antimicrobial agents. The resistance patterns were analyzed according to antimicrobial classes and bacterial classification (Gram-positive and Gram-negative). Monitoring antimicrobial susceptibility profiles is essential to support empirical treatment decisions and to guide antimicrobial stewardship strategies in hospital settings [23]. Antimicrobial susceptibility results were interpreted according to the Brazilian Committee on Antimicrobial Susceptibility Testing (BrCAST) guidelines, which are harmonized with the European Committee on Antimicrobial Susceptibility Testing (EUCAST) standards. Isolates were classified as susceptible, susceptible with increased exposure, or resistant according to the established clinical breakpoints applicable during the study period [24].

### 4.6. Variables

The main variables analyzed in the study included:

- Bacterial species identified in culture;
- Gram classification (Gram-positive or Gram-negative);
- Antimicrobial resistance profiles;
- Antimicrobial agents tested against each isolate;
- Antimicrobial classes evaluated in susceptibility testing.

These variables were used to analyze the microbiological profiles of the ICU and the resistance patterns of the isolated pathogens.

### 4.7. Statistical Analysis

Descriptive statistical analysis was performed to characterize the distribution of microorganisms and antimicrobial resistance patterns observed in the ICU during the study period. Categorical variables are summarized using absolute frequencies and percentages. Resistance rates were calculated for each antimicrobial agent tested against the bacterial isolates. The analysis also included the evaluation of resistance patterns according to bacterial classification and antimicrobial classes, enabling the identification of the most prevalent resistance profiles among Gram-positive and Gram-negative organisms. Missing data were handled using a complete-case approach. Isolates with unavailable susceptibility results for a given antimicrobial agent were classified as “not tested” (NT) and were excluded only from the calculation of resistance rates for that specific agent or antimicrobial class. No imputation was performed, since antimicrobial susceptibility was not missing at random but depended on the laboratory testing panel applied to each microorganism. All analyses were conducted using standard descriptive statistical methods for observational epidemiological studies. GraphPad InStat version 3.10 software (GraphPad Software, San Diego, CA, USA) was used [25,26].

### 4.8. Ethical Considerations

The study was conducted in accordance with ethical principles for research involving human data. Because the study used retrospective and anonymized laboratory and clinical records, no direct patient contact occurred. Data confidentiality and privacy were maintained throughout the research process. The study was approved by the Research Ethics Committee of the University of Sorocaba (CAAE 86145224.7.0000.5500). All data collection procedures ensured confidentiality, anonymity, and the possibility of study interruption at any time by the institution. As this study was based on secondary data obtained from medical records, the requirement for informed consent was waived in accordance with Brazilian regulations (Resolutions No. 466/2012 and No. 510/2016).

## 5. Conclusions

The present study demonstrates a high prevalence of multidrug-resistant microorganisms in the ICU setting, with a predominance of Gram-negative pathogens, particularly *Klebsiella pneumoniae* and *Acinetobacter baumannii*, exhibiting extensive resistance to multiple antimicrobial classes. Notably, a substantial proportion of the isolates belonged to the ESKAPE group, reinforcing the central role of these pathogens as major drivers of healthcare-associated infections and antimicrobial resistance in critically ill patients. The observed resistance patterns, especially to β-lactams, carbapenems, and quinolones, reflect a critical epidemiological scenario that significantly compromises empirical therapeutic strategies and limits available treatment options.

These findings highlight the urgent need for continuous local microbiological surveillance to support evidence-based clinical decision-making and optimize antimicrobial use. In addition, the high incidence and multidrug-resistant profiles of ESKAPE pathogens underscore the importance of strengthening antimicrobial stewardship programs and infection control strategies in intensive care units. Targeted interventions aimed at reducing selective pressure, improving antimicrobial prescribing practices, and preventing the dissemination of resistant organisms are essential to mitigate the impact of antimicrobial resistance and improve clinical outcomes in critically ill populations.

The novelty of this study lies in the integrated presentation of local ICU microbiology, pathogen-specific resistance patterns, and antimicrobial class-based resistance, providing a practical framework for empirical therapy review and institutional stewardship planning.

### Study Limitations

This study has some limitations that should be considered. As a retrospective, single-center analysis, the findings may not be fully generalizable to other hospitals or regions with different epidemiological profiles. In addition, the study was based mainly on microbiological laboratory data, which limited the evaluation of clinical severity, prior antimicrobial exposure, comorbidities, infection source, and patient-level outcomes. The absence of molecular testing prevented confirmation of specific resistance mechanisms, such as ESBL or carbapenemase genes. Moreover, some antimicrobial agents were not tested for all microorganisms, and these results were classified as not tested (NT), which may have affected the completeness of the resistance profiles. Finally, comparisons with external studies were descriptive and should be interpreted cautiously due to differences in study design, populations, laboratory methods, and surveillance periods.

## Figures and Tables

**Table 1:** Distribution of microorganisms isolated in the ICU (2024).

Microorganism	N	%
*Klebsiella pneumoniae*	287	15.33
*Staphylococcus haemolyticus*	164	8.76
*Enterococcus faecalis*	144	7.69
*Acinetobacter baumannii*	135	7.21
*Staphylococcus epidermidis*	117	6.25
*Pseudomonas aeruginosa*	114	6.09
*Staphylococcus hominis*	109	5.82
*Escherichia coli*	106	5.66
*Staphylococcus aureus*	99	5.29
*Candida albicans*	79	4.22
*Proteus mirabilis*	68	3.63
*Candida tropicalis*	48	2.56
*Candida glabrata*	43	2.30
*Enterococcus faecium*	43	2.30
*Staphylococcus capitis*	38	2.03
*Stenotrophomonas maltophilia*	34	1.82
*Enterobacter cloacae*	27	1.44
*Serratia marcescens*	16	0.85
*Enterobacter hormaechei*	12	0.64
*Haemophilus influenzae*	12	0.64
Total	1869	100.00

**Table 2:** Antimicrobial resistance profiles (%) of Gram-negative bacteria versus antimicrobial agents in the Intensive Care Unit, Brazil, 2025.

Microorganism	N	Gentamicin	(TMP-SMX)	AmiKacin	Amox-clav.	Aztreonan	Cefepime	Ceftazidime	Ceftazidime Avibactam	Ceftolozone Tazobactam	Ceftriaxone	Cefuroxime	Ciprofloxacin	Ertapenem	Meropenem	Piperacillin Tazobactam	Cephalexin	Norfloxacin
*Klebsiella pneumoniae*	287	55.3	57.1	32.6	88.1	92.8	91.1	90.1	13.2	81.2	93.93	93.9	91.3	70.8	67.6	86.5	95.4	93.4
*Acinetobacter baumannii*	135	48.3	81.7	91.3	NT	NT	NT	NT	NT	NT	NT	NT	96.6	NT	96.6	96.6	NT	NT
*Pseudomonas aeruginosa*	114	8.1	NT	4.1	NT	29.9	27.5	33	14.4	11.4	NT	NT	16.8	NT	21.2	39.3	NT	NT
*Escherichia coli*	106	19.5	31.7	5.8	40.5	65.3	47.6	52.8	0.0	6.2	54.1	58.6	58	0.0	0.0	17.6	43.2	46.1
*Proteus mirabilis*	68	65.5	66.6	9.5	56	41.8	40.6	23.2	26.7	30.9	69.8	72.5	63.4	28.5	28.5	0.0	100	85.7
*Enterobacter cloacae*	27	38.8	NT	10.5	0	57.8	47.7	57.8	0.0	31.5	63.16	60.0	47	36.84	5.2	58.8	NT	NT
*Serratia marcescens*	16	18.7	100.0	18.7	NT	14.2	25.0	14.2	0.0	7.1	25	0.0	25	12.5	12.5	20	NT	100.0
*Enterobacter hormaechei*	12	11.1	50.0	18.1	NT	55.5	36.3	55.5	11.1	60	60	0.0	36.3	27.2	9.0	63.6	NT	50.0

The color of the cells represents the resistance rates (%) of the microorganisms to the antimicrobial agents tested, ranging from red (higher resistance rates) to green (lower resistance rates). Intermediate colors represent proportional intermediate resistance rates. NT: Not tested. Trimethoprim–Sulfamethoxazole (TMP-SMX).

**Table 3:** Antimicrobial resistance profiles of Gram-positive bacteria versus antimicrobial agents in the Intensive Care Unit, Brazil, 2025.

Microorganism	N	Ampicillin	Levofloxacin	Linezolid	Teicoplanin	Vancomycin	Clindamycin	Erythromycin	Oxacillin	(TMP-SMX)
*Staphylococcus haemolyticus*	164	NT	90.8	0.0	0.0	0.0	92.2	98.5	97.8	73
*Enterococcus faecalis*	144	1.77	64.0	0.0	12.5	12.3	NT	NT	NT	NT
*Staphylococcus epidermidis*	117	NT	80.0	0.0	0.0	0.0	78.6	87.3	93.4	11.4
*Staphylococcus hominis*	109	NT	0.0	0.0	NT	0.0	0.0	0.0	0.0	0.0
*Staphylococcus aureus*	99	NT	12.2	0.0	0.0	0.0	46.9	63.8	40.9	9.6
*Enterococcus faecium*	43	100	100	0.0	100.0	100.0	NT	NT	NT	NT
*Staphylococcus capitis*	38	NT	87.5	0.0.	0.0	0.0	87.1	87.5	93.7	3.2

The color of the cells represents the resistance rates (%) of the microorganisms to the antimicrobial agents tested, ranging from red (higher resistance rates) to green (lower resistance rates). Intermediate colors represent proportional intermediate resistance rates. NT: Not tested; Trimethoprim–Sulfamethoxazole (TMP-SMX).

**Table 4:** Percentage of resistant strains by pharmacological class in an Intensive Care Unit (ICU), Brazil, 2025.

Microorganism/Pharmacological Class	N	Penicillins	Cephalosporins	Carbapenems	Aminoglycosides	Quinolones	Glycopeptides	Tetracyclines	Macrolides	Lipopeptides	Oxazolidinones	Rifamycins	Sulfonamides
*Acinetobacter baumannii*	135	96.6	NT	96.6	69.8	96.6	NT	NT	NT	NT	NT	NT	81.7
*Enterobacter cloacae*	27	29.4	43.3	21	24.7	47	NT	NT	NT	NT	NT	NT	NT
*Enterobacter hormaechei*	12	63.6	37.1	18.1	14.6	43.1	NT	NT	NT	NT	NT	NT	50.0
*Enterococcus faecalis*	144	1.7	NT	NT	65	64	12.4	0.0	NT	NT	0.0	NT	NT
*Enterococcus faecium*	43	87.0	NT	NT	55.7	100.0	20.2	0.0	NT	NT	2.9	NT	NT
*Escherichia coli*	106	29.1	37.4	0	12.7	52.0	NT	NT	NT	NT	NT	NT	31.7
*Klebsiella pneumoniae*	287	91.5	79.8	69.2	44	83.7	0	0	100	0	NT	0	57.1
*Proteus mirabilis*	68	28.4	51.9	28.5	37.5	74.6	NT	NT	NT	NT	NT	NT	66.6
*Pseudomonas aeruginosa*	114	39.3	21.6	21.2	6.1	16.8	NT	NT	NT	NT	NT	NT	NT
*Serratia marcescens*	16	20.0	11.9	12.5	18.7	62.5	NT	NT	NT	NT	NT	NT	100.0
*Staphylococcus aureus*	99	67.4	0.0	NT	1.23	12.2	0.0	0.0	63.8	0.0	0.0	0.0	9.6
*Staphylococcus capitis*	38	93.7	NT	NT	78.1	87.5	0.0	0.0	87.5	0.0	0.0	12.5	3.2
*Staphylococcus epidermidis*	117	93.4	NT	NT	49.4	80	0.0	0.0	87.3	0.0	0.0	21.3	11.4
*Staphylococcus haemolyticus*	164	97.8	NT	NT	87.2	90.8	0.0	0.0	98.5	0.0	0.0	72.3	73
*Staphylococcus hominis*	109	86.9	NT	NT	16.4	70.7	0.0	0.0	93.4	0.0	0.0	31.7	12.3

The color of the cells represents the resistance rates (%) of the microorganisms to the antimicrobial agents tested, ranging from red (higher resistance rates) to green (lower resistance rates). Intermediate colors represent proportional intermediate resistance rates. NT: Not tested.

## Data Availability

The data supporting the findings of this study are available from the authors upon reasonable request.
